# The Family Stress Model in families of children with rare diseases: a cross-sectional multilevel path analysis for understanding family dynamics

**DOI:** 10.3389/fpubh.2025.1713613

**Published:** 2025-11-18

**Authors:** Johannes Boettcher, Sarah Hohmann, Anne Daubmann, Jonas Denecke, Ania C. Muntau, Silke Wiegand-Grefe, Holger Zapf

**Affiliations:** 1 Department of Child and Adolescent Psychiatry, Psychotherapy and Psychosomatics, University Medical Center Hamburg-Eppendorf, Hamburg, Germany; 2 Department of Medical Biometry and Epidemiology, University Medical Center Hamburg–Eppendorf, Hamburg, Germany; 3 Department of Pediatrics, University Medical Center Hamburg-Eppendorf, Hamburg, Germany; 4 German Center for Child and Adolescent Health (DZKJ), Partner Site Hamburg, University Medical Center Hamburg-Eppendorf, Hamburg, Germany; 5 Department of Psychiatry and Psychotherapy, University Medical Center Hamburg-Eppendorf, Hamburg, Germany

**Keywords:** Family Stress Model, rare diseases, children, mental health, multilevel path analysis

## Abstract

**Background:**

Theoretical frameworks, such as the Family Stress Model, have been evaluated in various contexts; however, there is a lack of large-scale studies specifically focusing on families of children with rare diseases.

**Objective:**

To examine the applicability of the Family Stress model within a large-scale sample of families of children with rare diseases.

**Methods:**

The potential predictors of children’s mental health in a multicenter study of *n* = 872 parents of children with rare diseases were investigated. Factors contributing to children’s mental health within the Family Stress Model were investigated via cross-sectional multilevel path model.

**Results:**

Relevant associations were found among all variables. The multilevel model based on our data only partially supported the Family Stress Model. In our model, the quality of the parental relationship was not relevantly associated with stress and depressive symptoms.

**Conclusion:**

Our findings show that the Family Stress Model is not supported entirely in a multilevel analysis of families of children with rare diseases. Nevertheless, the results underscore the importance of focusing on alleviating parental stress, which could diminish intra-family psychopathology.

**Clinical trial registration:**

The study was registered on September 21, 2024, with the identifier number NCT04382820 at ClinicalTrials.gov.

## 1 Introduction

Caring for a child with a rare disease and the associated stressors introduces unique challenges that can significantly impact the psychological as well as relational well-being of both the parents and their children (1–3). In the context of families of children with rare diseases, theoretical frameworks like the Family Stress Model (4) may provide a valuable lens through which to examine how acute and chronic stressors may impact children’s adverse health outcomes through parental distress and interparental relationship problems (3). While the Family Stress Model has been utilized in different contexts and populations (5–7), a relevant gap remains in large-scale research focused on families with children who have rare diseases.

Rare diseases are characterized by their low prevalence, defined as conditions that impact fewer than 1 in every 2000 individuals (8). Rare diseases are often associated with a lack of accessible information, unpredictable progressions, and a shortage of specialized medical resources, all contributing to heightened stress for affected families (9). Additionally, families of children with rare diseases often experience both acute and chronic stressors, including financial hardship due to care management and supportive therapies, which can diminish parental productivity at work (10–12). Furthermore, the severity of a child’s disease and the perceived quality of life significantly influence stress levels, as parents of children with greater disease severity frequently encounter heightened emotional, physical, and financial burdens (13, 14). These stressors can influence psychological as well as relational well-being in parents, which in turn may have a dynamic impact on children’s behavioral problems within the family context (11). Considering these factors, examining them within a theoretical framework of stress seems to be particularly useful for investigating children’s adjustment problems in the context of rare diseases.

**Figure 1:**
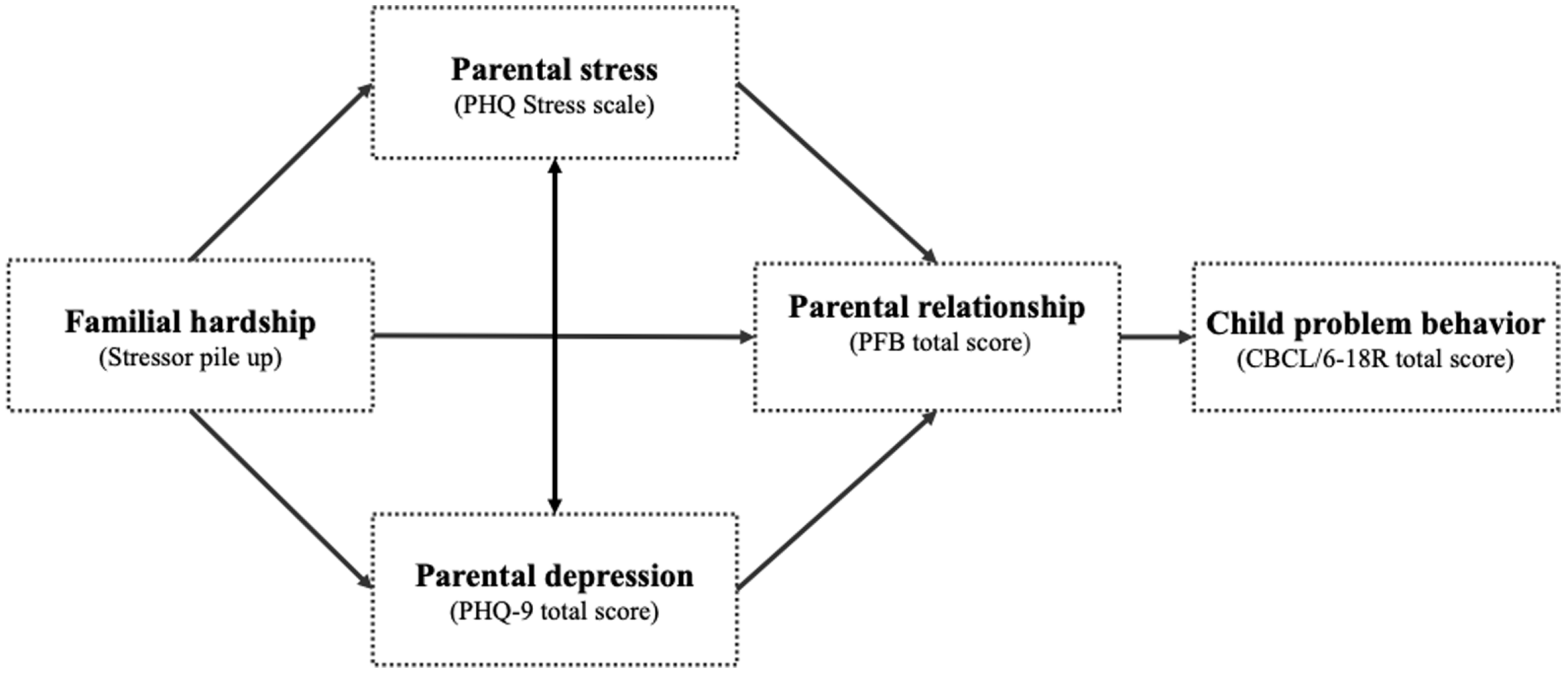
Adapted Family Stress Model (4).

The Family Stress Model (4) describes the process of children’s adjustment problems in response to acute or chronic stressors, taking into account various parental psychological and relational problems. The model begins with acute or chronic stressors, including economic and parental hardships (e.g., low income or challenges arising from the child’s special care needs). In the next step of the model, familial hardship leads to psychological distress in parents. These intermediary factors include perceived parental stress (e.g., feelings of being overwhelmed, difficulty managing daily tasks) and depressive symptoms (e.g., persistent sadness, loss of interest in activities). In the subsequent steps of the model, it is anticipated that parents’ psychological stress and depressive symptoms will heighten the likelihood of experiencing difficulties in their parental relationships. These parental conflicts (e.g., frequent arguments, lack of communication) exacerbate disruptions in parenting, which, in turn, culminate in child and adolescent maladjustment (e.g., behavioral problems, anxiety, depression). The result of these interactions is primarily defined as maladjustment in children and adolescents, which is often assessed in terms of mental health (15). Hereby, mental health can be defined as the “dynamic state of internal equilibrium which enables individuals to use their abilities in harmony with universal values of society” (16) (Figure 1).

Despite these theoretical advancements (3, 15) and empirical evidence of the Family Stress Model in different vulnerable historical crises (6) and populations (5, 7) there remains a notable gap of extensive research targeting families with children suffering from rare diseases. Therefore, current research sought to investigate factors within the Family Stress Model to investigate children’s mental health in response to acute or chronic stressors, with a specific aim to explore the fit of the model within our sample. In line with previous research (3), we defined (1) child problem behavior as children’s mental health, (2) parental relationship as perceived parental relationship satisfaction, (3) parent psychological distress as perceived stress and perceived symptoms of depression, and (4) familial hardship as a combination of economic, disease-specific, and parental hardships, collectively referred to as stressor pile-up. Additionally, we took the dependent nature of within-family relationships (between mothers and fathers) into account by including the family structure as a dependency in the model. The aforementioned recent advancements in statistical techniques for analyzing multilevel structural equation or path models (17) now enable researchers to explicitly examine and test indirect effects between groups.

Consistent with the Family Stress Model, higher levels of familial hardship were expected to be associated with greater levels of perceived stress (H1) and depression (H2) in parents, as well as lower parental relationship satisfaction (H3). Furthermore, familial hardship was anticipated to be indirectly associated to child problem behavior through diminished parental relationship satisfaction (H4).

## 2 Materials and methods

### 2.1 Study design

The initial data collected from parents of children diagnosed with rare diseases were examined as part of the CARE-FAM-NET study, a multicenter, rater-blinded, randomized controlled trial (18). This study was ethically approved by the Medical Chamber Hamburg (PV7161) and was preregistered at ClinicalTrials.gov (NCT04382820). In the current analysis, we use cross-sectional data from the baseline survey.

### 2.2 Measures

#### 2.2.1 Familial hardship

The variable of familial hardship was conceptualized as an additive stressor pile-up, with relevant factors selected based on literature review and discussions with clinicians about anticipated stressors. In line with prior research on stress theories, dichotomous variables were summed to represent the stressor pile-up (19). The stressor pile-up was calculated by summing the following nine dichotomous variables: parents’ absence of high school education (*n*[yes] = 303/872, 34.7%); parents’ absence of a higher education degree (*n*[yes] = 406/872, 46.6%); no current occupation (*n*[yes] = 63/872, 7.2%); desire to reduce working hours (*n*[yes] = 163/872, 18.7%); having a child with a progressive or life-threatening rare disease (e.g., neuromuscular diseases, oncological diseases) as adapted from Noeker (20) (*n*[yes] = 235/872, 26.9%); presence of a diagnosed physical disease in parents (*n*[yes] = 261/872, 29.9%); presence of a diagnosed mental disease in parents (*n*[yes] = 94/872, 10.8%); self-reported physical quality of life impairment (SF-12 physical component score ≥60) in parents (*n*[yes] = 85/872, 9.7%); and self-reported mental quality of life impairment (SF-12 mental component score ≥60) in parents (*n*[yes] = 22/872, 2.5%). These variables were selected to represent not only economic hardship that affects the family (21) but to represent also disease-specific hardship and lack of parental coping abilities as aspects of family hardship.

#### 2.2.2 Parental stress

The Patient-Health Questionnaire (PHQ) stress scale (22) was utilized to assess parental stress. The PHQ stress scale consists of ten items, each rated on a three-point scale from 0 (“not bothered”) to 2 (“bothered a lot”). The PHQ stress scale yields scores from 0 to 20, where higher scores indicate greater stress. The PHQ stress scale has been shown to have acceptable psychometric properties (22). The internal consistency was acceptable for the PHQ stress scale (*α* = 0.75).

#### 2.2.3 Parental depression

The Patient-Health Questionnaire-9 (23) was utilized to assess symptoms of depression in parents. The self-report instrument addresses key diagnostic criteria for major depressive disorder. The PHQ-9 comprises nine questions, each rated on a four-point scale from 0 (“not at all”) to 3 (“nearly every day”). The PHQ-9 yields scores from 0 to 27, where elevated scores indicate greater symptom severity. The PHQ-9 has been shown to have good psychometric properties (24). The internal consistency for the PHQ-9 sum score was good (*α* = 0.84).

#### 2.2.4 Parental relationship

The short form of the Partnership Questionnaire (“Partnerschaftsfragebogen-Kurzform”) (25) was utilized to assess parental relationship satisfaction. The instrument evaluates various aspects of relationship functioning, including quarrel behavior, tenderness, and togetherness/communication. The PFB-K comprises ten items, each rated on a four-point scale from 0 (“never/very rarely”) to 3 (“very often”). The total score is formed by summing the items, yielding scores from 0 to 30, with higher scores indicating better relationship satisfaction. The PFB-K has been shown to have good psychometric properties (25). The internal consistency for the PFB-K total score was acceptable (*α* = 0.70).

#### 2.2.5 Child adjustment

The Child Behavior Checklist/6-18R (CBCL/6-18R) (26) was used to measure child adjustment. The CBCL/6-18R is a parent-reported instrument designed to assess emotional and behavioral problems in children aged 6 to 18. The instrument comprises of 113 items; each rated on a three-point scale from 0 (“not true”) to 2 (“very true or often true”). In this study, we solely used T-values for the total score, with higher scores indicating a greater degree of emotional and behavioral problems. Since data were sometimes available for more than one child with a rare disease in a family, we included only the data from the CBCL/6-18R for the oldest diseased child available. The German version of the CBCL/6-18R has good psychometric properties (26). The internal consistency for the CBCL/6-18R total score was excellent (*α* = 0.96).

#### 2.2.6 Socio-demographic and clinical variables

Parents filled out a study-specific socio-demographic questionnaire that collected information about their sex, age, and socioeconomic status. The clinical variables related to the children encompassed various rare diseases, which were classified into broader groups according to the Orphanet classification system and Noeker’s classification (20). For psychosocial variables, parents provided self-reports regarding the presence of physical and mental health conditions, as well as their perceived physical and mental quality of life impairments, assessed using the Short Form 12 (SF-12) (27).

### 2.3 Sample

The current analysis utilized baseline data from the parents participating in the CARE-FAM-NET trial (18). All participating parents provided written informed consent and retained the right to withdraw from the study at any stage. The baseline dataset comprised information from 687 families with children and adolescents diagnosed with rare diseases, collected through the rater-blinded, randomized, controlled multicenter trial known as CARE-FAM-NET. Initially, 1,168 caregivers with were part of the baseline data. To account for the consistency in the analysis, only the data from biological parents were considered, while excluding data involving the stepmother or the partner of the father (*n* = 1), as well as the stepfather or the partner of the mother (*n* = 14), others (*n* = 10) and not stated (*n* = 8). In the next step, to account for the hierarchical structure and answer the research question, we included only datasets from parent pairs, excluding divorced fathers (*n* = 11) and mothers (*n* = 43), and widowed fathers (*n* = 0) and mothers (*n* = 5), and those not stating their relatedness (*n* = 4). Moreover, mothers (*n* = 178) and fathers (*n* = 15) where the partner did not take part in the study or were in a new partnership, were excluded. Ultimately, data for 436 families were retained, which included a total of 872 biological parents—comprising 436 mothers and 436 fathers. All participating parents had at least one child diagnosed with a rare disease, as defined by the European Commission (8). Prior to inclusion in the study, each diagnosis was verified by qualified medical professionals to ensure accuracy and validity.

### 2.4 Data analyses

Statistical analyses were conducted using cross-sectional data obtained from the multi-center trial CARE-FAM-NET. To address the problem of missing data in the measures, we employed Expectation Maximization (EM) imputation. EM imputation is recognized for its efficacy in dealing with incomplete datasets, particularly within the framework of structural equation modeling (28), as it produces complete datasets by explicitly estimating and imputing missing values. Participants who did not respond were removed from the analysis if any of their variables exhibited over 30% missing data (*n* = 0), as the EM algorithm functions optimally when the missing data is below this threshold. As a result, we could proceed with the final sample of 872 parents. Missing data varied across variables: Familial hardship (3.7%), parental stress (0.3%), parental depression (0.0%), parental relationship (5.3%), and child adjustment (0.8%). The additional conducted Little’s MCAR test was not statistically significant, *χ*^2^(31) = 43.73, *p* = 0.064, indicating that the data were missing completely at random.

The data analysis involved the application of descriptive statistics, which encompassed the calculation of frequencies, means, and standard deviations. To explore the association among the variables outlined in the Family Stress Model, we utilized Pearson correlation analyses. To account for the nested data structure, a multilevel path model was conducted using the package *lavaan* (29). The model was specified to simultaneously estimate direct and indirect relationships among the variables at both the within-family and between-family levels. The fit of the models was evaluated using standard measures for both absolute and relative goodness of fit (30): The Root Mean Square Error of Approximation (RMSEA), which is considered an absolute measure of fit with a threshold of <0.08, is indicative of a strong alignment between the model specification and the observed data. The Comparative Fit Index (CFI) and the Tucker-Lewis Index (TLI), which are considered as relative measures of goodness of fit with a cut-off of ≥0.90, are used to indicate a good fit. The Standardized Root Mean Square Residual (SRMR), which is considered an absolute fit index with a threshold of < 0.10 is considered a good fit. We additionally conducted sensitivity analyses of the models using the estimator (FIML), which confirmed that the robustness of our findings was maintained across different missing data handling methods. The hypothesized associations between constructs were evaluated using both direct and indirect effects, which were reported as unstandardized path coefficients (*β*) along with their 95% confidence intervals (95% CI). The threshold for statistical significance was set at *p* < 0.05 (two-tailed) and was utilized in a descriptive context. To assess the proportion of variance attributable to family-level clustering, intraclass correlations (ICCs) were estimated using unconditional random-intercept models (lme4 package in R). We also calculated single level path models separated by role (mother/father) to contrast the results with the multilevel model. Statistical analyses were conducted using R Statistical Software [v2024.04.2 + 7, R Core Team (31)].

## 3 Results

Table 1 presents the main sociodemographic and disease-specific characteristics for families of children with rare diseases.

**Table 1:** Sociodemographic and clinical characteristics of parents of children with rare diseases.

Parents (*n* = 872)	M	SD
Age (years)		
Female (*n* = 436)	38.7	6.41
Male (*n* = 436)	41.3	7.14
Number of children in family	2.0	0.82

	*n*	%
Marital status (mothers/fathers)		
Unmarried with life partner and	66/30	15.2/6.9
Married	370/367	84.9/84.2
Not stated	0/8	0.0/1.8
Highest school qualification (mothers/fathers)		
Without qualification	1/1	0.2/0.2
Primary school	22/55	5.0/12.6
Secondary school	113/111	25.9/25.4
High school diploma	269/230	61.7/52.8
Other qualification	8/4	1.8/0.9
Not stated	23/35	5.3/8.0
Highest professional qualification (mothers/fathers)		
Without qualification	9/14	2.1/3.2
Apprenticeship	137/145	31.4/33.3
Master craftsman training	42/60	9.6/13.8
Technical college/University	196/172	45.0/39.4
Other qualification	13/19	3.0/4.4
Not stated	39/26	8.9/5.9
No current occupation (mothers/fathers)	38/24	8.7/5.5
Desire to reduce working hours (mothers/fathers)	56/107	12.8/24.5
Current diagnosed physical disease (mothers/fathers)	140/120	32.1/27.5
Current diagnosed mental disease (mothers/fathers)	56/36	12.8/8.3

Children and adolescents with rare diseases (*n* = 436)	M	SD
Age (years)		
Female (*n* = 188)	8.1	5.21
Male (*n* = 248)	6.7	4.38

Clinical variables	*n*	%
Disease groups according to Noeker (20)		
Diseases with an episodic-relapsing course	46	10.6
Diseases with permanent functional limitations & disability	272	62.4
Diseases with a progressive or life-threatening course	118	27.1

M, mean; SD, standard deviation.

### 3.1 Correlation analyses

Table 2 illustrates the associations among the variables of the Family Stress Model, as indicated by Pearson correlation coefficients and their corresponding 95% confidence intervals. Relevant bivariate associations were found for all variables, with effect sizes ranging from low to high. Additionally, correlation coefficients for mothers and fathers are provided separately in Supplementary Table 2. Gender-specific investigations of associations between the variables revealed relevant bivariate associations for all variables, except for the associations between child emotional and behavioral problems and parental stress, and between child emotional and behavioral problems and parental depression in fathers.

**Table 2:** Pearson correlation between predictor and outcome parameters.

Variables	1	2	3	4	5
Parents (*n* = 872)					
1. Stressor pile-up	–				
2. Parental stress (PHQ stress scale)	**0.275 [0.212, 334]**	–			
3. Parental depression (PHQ-9)	**0.286 [0.224, 0.345]**	**0.705 [0.670, 0.737]**	–		
4. Parent relationship (PFB-K)	**−0.122 [−0.189, −0.055]**	**−0.381 [−0.438, −0.321]**	**−0.263 [−0.325, −0.198]**	–	
5. Child problem behavior (CBCL/6-18R)	**0.117 [0.050, 0.182]**	**0.158 [0.092, 0.223]**	**0.116 [0.050, 0.181]**	**−0.142 [−0.208, −0.075]**	–
*M*	2.0	5.2	5.8	20.4	45.9
*SD*	1.09	3.67	4.50	5.13	16.6
*Median*	2	4	5	21	37
*Min, max*	0, 6	0, 18	0, 23	4, 30	31, 91

M, mean; SD, standard deviation; Min, minimum score; Max, maximum score. Main entries are Pearson r, with 95% confidence intervals values in parenthesis. PHQ stress scale, Patient Health Questionnaire stress scale; PHQ-9, Patient Health Questionnaire-9; PFB-K, Partnerschaftsfragebogen-Kurzform; CBCL/6-18R, Child Behavior Checklist for ages 6–18, revised.The bold values indicate statistically significant results (*p* < 0.05).

### 3.2 Multilevel path analysis

**Figure 2:**
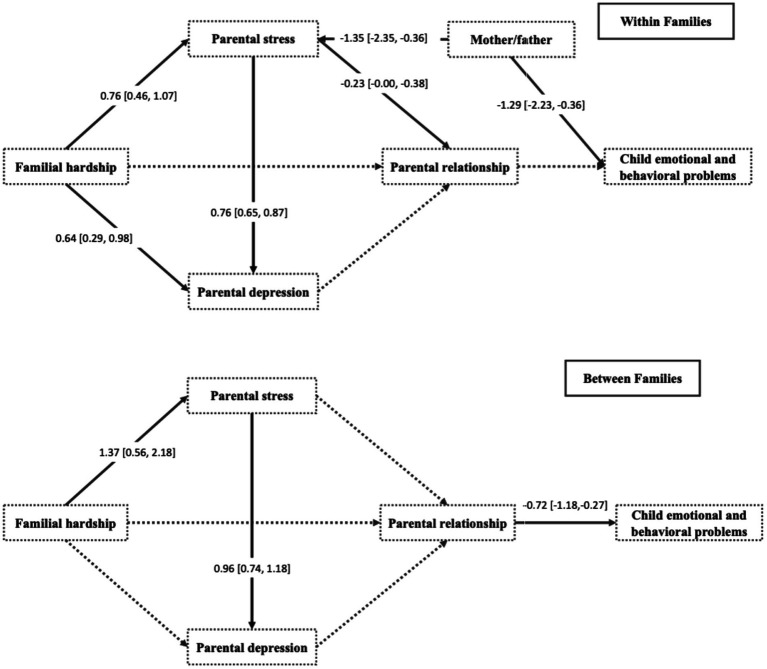
Multilevel path analysis models.

The theory-driven multilevel path analysis was performed to evaluate the interrelationship among the variables. The results are presented in Figure 2. The model showed a sufficiently good fit CFI = 1.000, TLI = 1.034, RMSEA = 0.000, and SRMR = 0.076 with *χ^2^*(6) = 0.000 and *p* = 1.000.

Within-family analyses revealed that higher parental stress was significantly associated with lower parental relationship satisfaction (*β* = −0.23, 95% CI: [−0.38, −0.08], *p* < 0.001). Parental depression symptoms were not significantly associated with parental relationship satisfaction at the within- family level (*β* = −0.02, *p* = 0.710). Familial hardship did not show a significant association with parental relationship satisfaction (*β* = −0.20, *p* = 0.270). Parental relationship satisfaction was not significantly associated with child emotional and behavioral problems (*β* = −0.12, *p* = 0.251). At the between-family level, familial hardship was significantly associated with parental stress (*β* = 1.37, 95% CI: [0.56, 2.18], *p* = 0.001), and parental stress was significantly associated with parental depression symptoms (*β* = 0.96, 95% CI: [0.74, 1.18], *p* < 0.001). Parental relationship satisfaction was negatively associated with child emotional and behavioral problems (*β* = −0.72, 95% CI: [−1.18, −0.27], *p* = 0.002). For all results cf. Supplementary Table 2.

The ICCs indicated that 59.4% of the variance in parental relationship satisfaction, 15.2% in parental depression symptoms, 26.6% in parental stress, and 89.9% in child emotional and behavioral problems were located at the between-family level. These results justify the use of a multilevel structural equation modeling (MSEM) framework to account for the nested data structure.

In the additional analyses separated by parental role (single analyses for mothers and fathers), model fit was satisfactory for fathers (CFI = 0.997, RMSEA = 0.033, SRMR = 0.023) and acceptable for mothers (CFI = 0.970, RMSEA = 0.094, SRMR = 0.048). Across both groups, the hypothesized stress-related pathway from parental stress to parental relationship was consistently negative, whereas in the model for fathers, the path between familial hardship and parental depression was additionally relevant (see Supplementary Figure 1).

## 4 Discussion

While the importance of family adaptation in managing rare diseases is acknowledged, the exploration of familial factors within a theoretical framework remains insufficiently addressed (13, 32). Additionally, sample sizes for specific diseases are often too small to support substantial conclusions (33, 34). To address these research gaps concerning families of children with rare diseases, the present study employed the Family Stress Model (4) as a theoretical foundation to examine the relationships among familial hardship, parental stress, parental depression symptoms, parental relationship satisfaction, and child emotional and behavioral problems, utilizing baseline data from a multicenter trial.

With our data, the paths expected according to the Family Stress Model were only partly confirmed in the context of parents of children with rare diseases. The excellent global fit of the multilevel model should be interpreted in light of the substantial clustering observed in the data. The intraclass correlations indicated that a large proportion of variance in family functioning and child adjustment was located at the between-family level (ICC = 0.59 for PFB and 0.90 for CBCL), whereas parental stress and depressive symptoms showed lower but meaningful clustering (ICCs = 0.27 and 0.15, respectively). This pattern suggests that the primary associations among parental well-being, family processes, and child outcomes emerge between rather than within families, which also explains the non-significant within-level paths despite high overall model fit.

Our multilevel model showed that on the between-family level, the relation between familial hardship and parental depression was mediated by parental stress, and parental relationship quality predicting the perception of child emotional and behavioral problems. These findings support previous studies that demonstrate an association between familial hardship, in the form of both current and chronic stressors, and symptoms of parental depression (35, 36). These findings also support the idea that increased exposure to cumulative stressors may lead to perceived stress and depressive symptoms in both mothers and fathers. With regard to the association of perceived parental relationship quality and child emotional and behavioral problems, the results of the model are in line with previous research (37, 38).

On the within family level, the parents that report higher familial hardship also report more parental stress and more parental depression. Also, higher parental stress predicted parental depression and lower relationship quality. Being a father decreased perceived parental stress and child emotional and behavioral problems. Mothers, who often act as primary caretakers, reported more parental stress and child emotional and behavioral problems, which supports findings from earlier studies on parents of children with rare diseases (33), neurodevelopmental disorders (39), chronic diseases (35), and the general adult population (40, 41). These gender-specific influences may arise from societal and cultural norms that designate mothers as primary caregivers, leading to greater involvement in their children’s care and a heightened emotional response to their children’s disease (35).

The multilevel model could not establish a path between family hardship and parental relationship on the between-family level. A reason might be that the burden associated with caring for a child with a rare disease may be processed as something meaningful and leading to a higher sense of coherence, which may mitigate effects of hardship on the parental relationship (47). In our additional single-level analyses for mothers and fathers separately, additional paths became significant in the model. These findings indicate that structural associations are robust across informants while the more complex model that respects the hierarchical structure of the data is more restrictive and less likely to confirm the hypothesized model because standard errors are estimated more realistically and variance is distributed between the within and the between levels (42). Our findings highlight the critical role of intervening factors in understanding parental stress in families of children with rare diseases. Therefore, within the context of the Family Stress Model, it was established that stress may function as a central factor. Future interventions should focus on stress reduction by integrating proven elements of approaches such as cognitive-behavioral therapy (e.g., Dialectical Behavior Therapy) or relaxation programs (e.g., Mindfulness-Based Stress Reduction) (43), with emotion regulation as the overarching framework for stress management (41), given that emotion regulation is recognized as a fundamental treatment approach across various disorders (44).

### 4.1 Strengths and limitations

The study presents several commendable features. We obtained data prospectively from extensive multicenter samples and employed robust statistical analyses accounting for the hierarchical structure of the data within a theoretical framework. Additionally, the study was preregistered in international databases prior to the beginning of data collection. Despite the valuable findings, several limitations must be acknowledged. Firstly, the diverse nature of the rare diseases included in this study—ranging from episodic-relapsing types to progressive or life-threatening conditions—could complicate the interpretation of the results across different disease categories. Nevertheless, to account for the heterogeneity of the diseases, we incorporated disease severity into the variable familial hardship. By including an overarching factor, we were able to develop a comprehensive overall model. While certain rare disease groups were adequately represented, others were underrepresented, which may limit the generalizability of our results to broader classifications. Secondly, a selection bias may exist among the parents involved in the study. Families who participate in rare disease research might differ systematically from those who do not, leading to a possible overrepresentation of more proactive or educated individuals. This disparity could restrict the applicability of the findings to the wider population of families affected by rare diseases (45). Thirdly, our analyses are based on self-report measures. Integrating external assessments and behavior observations would increase the reliability of the data. Fourth, while the use of equally weighted dichotomous indicators for the stressor pile-up index facilitates simplicity and interpretability, especially within the constraints of our cross-sectional data, alternative approaches such as data-driven weighting methods may offer advantages. For example, although beyond the scope of our current study, Item Response Theory can improve the validity and measurement precision of composite scores through the estimation of optimal weights, which could benefit future research (46). Last but not least, although our cross-sectional path analysis suggests a framework for considering the accumulation of stress, it does not provide direct evidence to substantiate causal relationships. As a result, additional research employing the Family Stress Model in a longitudinal context is crucial for thoroughly investigating psychosocial outcomes and identifying potential intervention targets for families of children with rare diseases across different developmental stages.

## 5 Conclusion

Although the Family Stress Model has been applied to different contexts and populations (5–7), it has not yet been used in a large cohort of families of children with rare diseases. Our findings contribute to the literature on parental adaptation by emphasizing the familial dynamics specific to families of children with rare diseases within this theoretical framework. The results indicate that customized emotion regulation programs designed to alleviate parental stress could help diminish intra-family psychopathology. Future research should focus on longitudinal studies to further clarify the factors that facilitate effective familial adjustment.

## Data Availability

The raw data supporting the conclusions of this article will be made available by the authors upon reasonable request.
