# Supplementary material for: The Family Stress Model in families of children with rare diseases: a cross-sectional multilevel path analysis for understanding family dynamics
Source: Front Public Health. 2025 Nov 18;13:1713613. doi: 10.3389/fpubh.2025.1713613 (PMC12671385; doi:10.3389/fpubh.2025.1713613)
Supplement: Supplementary file 1 [file Supplementary_file_1.docx]

| **Supplementary Table 1.** Pearson correlation between predictor and outcome parameters in both mothers and fathers. | | | | | |
| --- | --- | --- | --- | --- | --- |
| Variables | 1 | 2 | 3 | 4 | 5 |
| Mothers (*n* = 436) |  |  |  |  |  |
| 1. Stressor pile-up | - |  |  |  |  |
| 2. Parental stress (PHQ stress scale) | **.278 [.189, .362]** | - |  |  |  |
| 3. Parental depression (PHQ-9) | **.245 [.154, .331]** | **.648 [.590, .699]** | - |  |  |
| 4. Parent relationship satisfaction (PFB-K) | **-.102 [-.197, -.006]** | **-.443 [-.518, -.362]** | **-.299 [-.384, -.208]** | - |  |
| 5. Child problem behavior (CBCL/6-18R) | **.114 [.020, .206]** | **.214 [.122, .302]** | **.152 [.059, .243]** | **-.141 [-.235, -.045]** | - |
| *M* | 2.0 | 5.9 | 6.7 | 20.6 | 46.5 |
| *SD* | 1.09 | 3.73 | 4.61 | 5.19 | 17.22 |
| *Median* | 2 | 5 | 6 | 21 | 39 |
| *Min, max* | 0, 6 | 0, 18 | 0, 23 | 5, 30 | 31, 91 |
| Fathers (*n* = 436) |  |  |  |  |  |
| 1. Stressor pile-up | - |  |  |  |  |
| 2. Parental stress (PHQ stress scale) | **.282 [.193, .366]** | - |  |  |  |
| 3. Parental depression (PHQ-9) | **.343 [.257, .423]** | **.749 [0.705, 0.788]** | - |  |  |
| 4. Parent relationship satisfaction (PFB-K) | **-.142 [-.230, -.047]** | **-.346 [-.428, -.258]** | **-.252 [-.339, -.159]** | - |  |
| 5. Child problem behavior (CBCL/6-18R) | **.120 [.026, .212]** | .085 [-.010, .178] | .060 [-.035, .154] | **-.146 [-.239, -.051]** | - |
| *M* | 1.9 | 4.5 | 4.9 | 20.2 | 45.2 |
| *SD* | 1.09 | 3.48 | 4.19 | 5.06 | 15.95 |
| *Median* | 2 | 4 | 4 | 21 | 38 |
| *Min, max* | 0, 6 | 0, 17 | 0, 20 | 4, 30 | 31, 88 |
| *Note.* *M =* Mean, *SD =* Standard deviation*, Min =* minimum score*, Max = maximum score.* Main entries are Pearson *r*, with 95% confidence intervales values in parenthesis. PHQ stress scale = Patient Health Questionnaire stress scale, PHQ-9 = Patient Health Questionnaire-9, PFB-K = Partnerschaftsfragebogen-Kurzform, CBCL/6-18R = Child Behavior Checklist for ages 6-18, revised. | | | | | |
