# Supplementary material for: The Family Stress Model in families of children with rare diseases: a cross-sectional multilevel path analysis for understanding family dynamics
Source: Front Public Health. 2025 Nov 18;13:1713613. doi: 10.3389/fpubh.2025.1713613 (PMC12671385; doi:10.3389/fpubh.2025.1713613)
Supplement: Supplementary file 2 [file Supplementary_file_2.docx]

**Supplementary Table 2.** Standardized and unstandardized parameter estimates (95% CIs based on sandwich standard errors).

| **Outcome** |  |  |  |  |  |
| --- | --- | --- | --- | --- | --- |
| ***Within family paths*** | **Predictor** | **B** | ***β*** | **95% CI** | ***p* value** |
| Parental relationship | Parental stress | −0.230 | −0.216 | [−0.376, −0.084] | .002 |
| Parental relationship | Parental depression | −0.019 | −0.024 | [−0.121, 0.083] | .712 |
| Parental relationship | Stressor Pile-Up | −0.199 | −0.056 | [−0.553, 0.155] | .270 |
| Parental relationship | Parental role | −0.732 | −0.112 | [−1.611, 0.147] | .103 |
| Parental depression | PileUp | 0.637 | 0.146 | [0.294, 0.980] | <.001 |
| Parental depression | Parental stress | 0.764 | 0.581 | [0.653, 0.874] | <.001 |
| Parental stress | Stressor Pile-Up | 0.764 | 0.230 | [0.462, 1.065] | <.001 |
| Parental stress | Parental role | −1.351 | −0.220 | [−2.346, −0.355] | .008 |
| Child emot. problems | Parental relationship | −0.120 | −0.075 | [−0.325, 0.084] | .250 |
| Child emot. problems | Parental role | −1.294 | −0.123 | [−2.227, −0.361] | .007 |
| ***Between family paths*** | **Predictor** | **B** | **β** | **95% CI** | ***p* value** |
| Parental relationship | Parental stress | −2.777 | −1.406 | [−9.100, 3.546] | .389 |
| Parental relationship | Parental depression | 1.523 | 0.751 | [−4.845, 7.890] | .639 |
| Parental relationship | Stressor Pile-Up | 1.013 | 0.147 | [−1.943, 3.969] | .502 |
| Parental depression | Parental stress | 0.960 | 0.985 | [0.743, 1.176] | <.001 |
| Parental stress | Stressor Pile-Up | 1.369 | 0.393 | [0.557, 2.182] | .001 |
| Child emot. problems | Parental relationship | −0.722 | −0.182 | [−1.178, −0.267] | .002 |
| **Indirect effects** |  |  |  |  |  |
| ***Indirect Path*** |  |  | **Estimate** | **95% CI** | ***p* value** |
| Stressor Pile-Up → Parental stress → Parental relationship  Parental role → Parental stress → Parental depression | | | −0.176 | [−0.315, −0.063] | .004 |
|  |  |  | −1.030 | [−1.800, −0.420] | .001 |
