# Supplementary material for: The Family Stress Model in families of children with rare diseases: a cross-sectional multilevel path analysis for understanding family dynamics
Source: Front Public Health. 2025 Nov 18;13:1713613. doi: 10.3389/fpubh.2025.1713613 (PMC12671385; doi:10.3389/fpubh.2025.1713613)
Supplement: Supplementary file 3 [file Supplementary_file_3.docx]

|  |
| --- |
| **** |
| **Supplementary Figure 1.** Path analysis according to the Family Stress Model in both mothers and fathers. |
